# Novel Hydrogen Hydrate Structures under Pressure

**DOI:** 10.1038/srep05606

**Published:** 2014-07-08

**Authors:** Guang-Rui Qian, Andriy O. Lyakhov, Qiang Zhu, Artem R. Oganov, Xiao Dong

**Affiliations:** 1 Department of Geosciences, Stony Brook University, Stony Brook, New York 11794-2100, USA; 2 Department of Geosciences, Center for Materials by Design, and Institute for Advanced Computational Science, State University of New York, Stony Brook, NY 11794-2100; 3 Moscow Institute of Physics and Technology, 9 Institutskiy lane, Dolgoprudny city, Moscow Region 141700, Russia; 4 School of Materials Science, Northwestern Polytechnical University, Xi'an 710072, China; 5 School of Physics and MOE Key Laboratory of Weak-Light Nonlinear Photonics, Nankai University, Tianjin 300071, China

## Abstract

Gas hydrates are systems of prime importance. In particular, hydrogen hydrates are potential materials of icy satellites and comets, and may be used for hydrogen storage. We explore the H_2_O–H_2_ system at pressures in the range 0–100 GPa with *ab initio* variable-composition evolutionary simulations. According to our calculation and previous experiments, the H_2_O–H_2_ system undergoes a series of transformations with pressure, and adopts the known open-network clathrate structures (sII, C_0_), dense “filled ice” structures (C_1_, C_2_) and two novel hydrate phases. One of these is based on the hexagonal ice framework and has the same H_2_O:H_2_ ratio (2:1) as the C_0_ phase at low pressures and similar enthalpy (we name this phase *Ih*-C_0_). The other newly predicted hydrate phase has a 1:2 H_2_O:H_2_ ratio and structure based on cubic ice. This phase (which we name C_3_) is predicted to be thermodynamically stable above 38 GPa when including van der Waals interactions and zero-point vibrational energy, and explains previously mysterious experimental X-ray diffraction and Raman measurements. This is the hydrogen-richest hydrate and this phase has a remarkable gravimetric density (18 wt.%) of easily extractable hydrogen.

Molecular compounds (cocrystals) of water ice (H_2_O) and hydrogen (H_2_) are known to form clathrate structures with hydrogen molecules encapsulated as guests in the host sublattice formed by water molecules. Hydrogen hydrates, as environmentally clean and efficient hydrogen storage materials, have excited significant interest. Extensive literature exists from both experimental^1^,^2^,^3^,^4^,^5^,^6^,^7^,^8^,^9^,^10^,^11^,^12^ and theoretical^13^,^14^ sides. Aside from the H_2_ molecules, many other small molecules can be encapsulated as guests in clathrates at elevated pressure (e.g., noble gases, nitrogen, oxygen, methane, chlorine). (See Ref. [15] and references therein) Hydrogen hydrates are important as potentially major materials of icy satellites and comets, and hydrogen storage materials.

Twenty years after the first report of the formation of two filled-ice hydrogen hydrates by Vos *et al.*^1^, four hydrogen hydrate forms are known to exist at elevated pressures. Two of the hydrogen hydrates are clathrates, denoted as clathrate structure II (sII)^3^,^5^ and compound 0 (C_0_)^12^, the other two are filled ice hydrates, compound 1 (C_1_) and compound 2 (C_2_)^1^,^2^. The sII clathrate hydrate was synthesized under pressures of 180 to 220 MPa at 300 K, and its structure was shown to contain 48 hydrogen molecules and 136 water molecules in the unit cell^3^. The C_0_ clathrate was recently found to be stable near 0.5 GPa and to have the composition 2H_2_O:1H_2_ and a trigonal quartz-like structure^12^. The water molecules in the C_0_ structure are arranged in a totally new way, different from the known ices or ice sublattices in hydrates structures. This structure has space group *P*3_2_21, but this could possibly go as low as *P*3_2_, depending on how the hydrogens are arranged^12^.

At higher pressures, clathrates give way to denser structures of the filled ice type. The C_1_ and C_2_ phases are formed at 0.36–0.9 GPa and ~2.4 GPa, respectively^1^,^2^,^11^. The C_1_ hydrate has a water host framework based on ice-II and a 6:1 water to hydrogen ratio. C_2_ has a 1:1 ratio of water to hydrogen and is composed of water molecules in the “cubic ice” (ice-Ic) framework and rotationally disordered hydrogen molecules^15^. Recent experiments^7^,^8^,^9^,^10^ indicate that the C_2_ hydrate undergoes a structural transformation from cubic to tetragonal phase at around 10–20 GPa, with an increasing difference in the unit cell axes, and then transforms to another high-pressure phase near ~45 GPa. This high-pressure phase is maintained up to at least 80 GPa but its structure is not fully resolved. Given the difficulties in characterization of the chemical composition and crystal structure of these hydrates, and believing that new phases are likely to exist, we decided to perform a computational search to revisit the H_2_O–H_2_ system under pressure.

## Results

**Figure 1:**
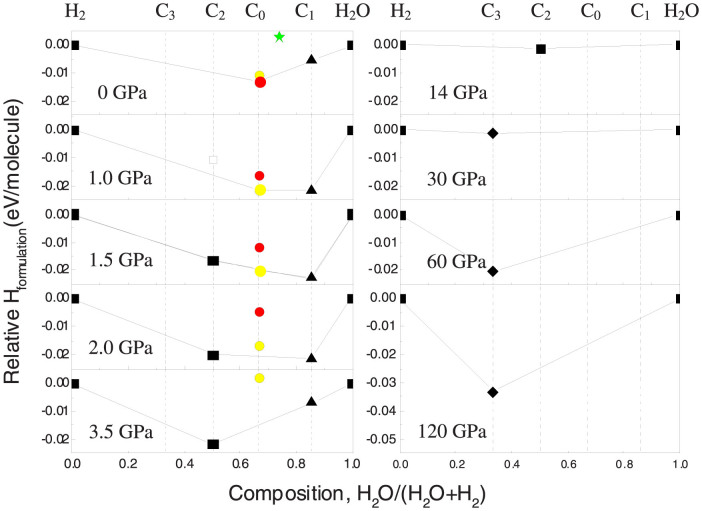
Convex hull diagram for H_2_O-H_2_ system at selected pressures and zero temperature. This figure shows the enthalpy of formation (in eV/molecule) of molecular compounds from pure H_2_O ice and H_2_. The red and yellow circles represent the C_0_ and *Ih*-C_0_ phases, respectively. The green star represents the sII structure.

Using the evolutionary algorithm USPEX^16^,^17^,^18^,^19^,^20^, we explored all possible stable phases in the H_2_O–H_2_ system (See *Methods*). Remarkably, we have found two novel filled ice hydrogen hydrates, and all known hydrogen hydrates (except the sII structure, because of the very large number of molecules in its unit cell). Thus, at pressures in the range 0–2 GPa, the sII structure was input separately in order to calculate stability ranges of phases in the H_2_O-H_2_ system. Fig. 1 shows the convex hull diagram for the H_2_O-H_2_ system.

**Figure 2:**
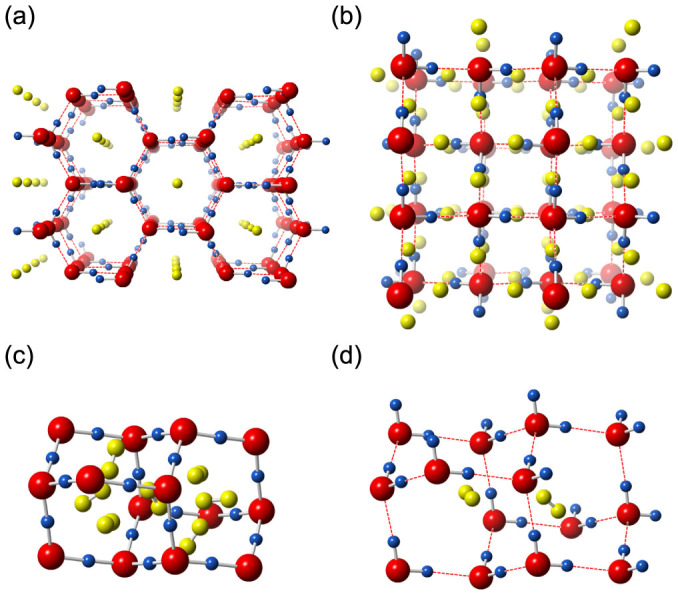
Crystal structures of *Ih*-C_0_ and C_3_ phases, cage structures in C_2_ and C_3_ phases. (a) *Ih*-C_0_ structure at 0.5 GPa, (b) C_3_ structure at 30 GPa, (c) cages formed by water molecules in C_3_ at 100 GPa, the hydrogen molecules are located at the center of each chair-like H-O ring, (d) cages in “filled ice-Ic” C_2_, hydrogen molecules are in the center of the cage. Large red and small blue spheres are O and H atoms in water molecules, respectively; the yellow spheres represent the H_2_ molecules in (a) and (b), and represent H atoms in (c) and (d). Red dashed lines represent hydrogen bonds.

With including the van der Waals (vdW) dispersion forces (See *Methods*), our results are generally in very good agreement with experiments, but with several novel aspects. At 0 GPa, the C_0_, C_1_ and a novel hydrogen hydrate phase are found stable or nearly stable in the H_2_O–H_2_ system, while the sII phase is metastable (~0.013 eV/molecule less stable than the mixture of stable compounds C_0_ and C_1_). The structure of the novel hydrogen hydrate is based on the framework of hexagonal ice (ice-Ih), with two hydrogen molecules hosted inside channels running along the hexagonal axis (Fig. 2a). It has a 2:1 ratio of water to hydrogen, same as C_0_, and space group *Cc*. We name it *Ih*-C_0_ to distinguish from C_0_. The enthalpy of the *Ih*-C_0_ phase is close to C_0_, and is slightly lower at pressures above ~0.4 GPa (Fig. S2 in Supplementary Information). At 1.5 GPa, in addition to the C_0_, *Ih*-C_0_ and C_1_ phases, the hydrate phase C_2_ with an ice-Ic framework structure becomes stable.

At pressures above 2 GPa, the C_0_ and *Ih*-C_0_ phases are calculated to be above the convex hull, indicating that these phases become unstable against decomposition into C_1_ and C_2_. Above 3.5 GPa, the C_1_ phase will also become unstable, and the C_2_ phase will remain the only stable hydrate. For hydrate C_2_, USPEX calculations uncovered at least four typical energetically favorable candidate structures at different pressures, *P*4_1_2_1_2, *I*4_1_/*amd*, *Pna*2_1_ and *I*4_1_*md* (Fig. S3. in Supplementary Information). These structures differ in the orientations of water and hydrogen molecules, which is similar to Ref. [15]. The C_2_ phase will lose stability at ~14 GPa, which is much lower than 40 GPa suggested in the previous study^7^,^10^. We explain this by metastable persistence of C_2_ up to the pressure of 40 GPa. Between 14–28 GPa, there are, unexpectedly, no thermodynamically stable hydrates.

Near 30 GPa, another novel H_2_O–H_2_ phase is found to be stable at zero temperature. It has a 1:2 water to hydrogen ratio, and net composition H_6_O. This novel hydrogen hydrate, which we name C_3_, has the highest hydrogen concentration among all hydrogen hydrates. If it can be synthesized at low pressures, it would be an attractive hydrogen storage material, having 18 wt.% concentration of easily separable (non-water) hydrogen. The C_3_ structure has space group *P*4_1_ and is also based on the framework of ice-Ic (Fig. 2b), similar to low-pressure hydrate C_2_. The unit cell of C_3_ contains four water molecules, the H_2_ molecules are located at the center of chair-like H-O rings (formed by six oxygen and six hydrogen atoms) that form faces of the cage, as shown in Fig. 2c. Differently, in the C_2_ hydrate, the H_2_ molecules are in the center of the water cages (Fig. 2d). According to our calcualtions, the C_3_ phase will remain stable up to at least 120 GPa.

Our theoretical calculations indicate that the H_2_O–H_2_ system contains several stable phases, including open-network clathrate structures (C_0_) and dense filled ice phases (*Ih*-C_0_, C_1_, C_2_ and C_3_). The C_0_ phase is predicted to be stable at pressures below 1.5 GPa, which is close to the experimental result (below 0.8 GPa^12^). The C_1_ phase is predicted to be stable at pressures below 3.5 GPa, also close to the experimentally determined transition pressure of 2.5 GPa^1^. The zero-point vibration energy (ZPE) significantly affects the relative stability of hydrogen-rich structures^21^. We have estimated the ZPE within the quasi-harmonic approximation^22^ to refine the stability ranges of C_2_ and C_3_ phases above 10 GPa. When considering the ZPE, the stability field of the C_2_ phase expands up to ~19 GPa, but this phase remains dynamically stable, and thus can exist as a metastable material at pressures of at least 60 GPa (Fig. S9 and Fig. S10 in Supplementary Information).

**Figure 3:**
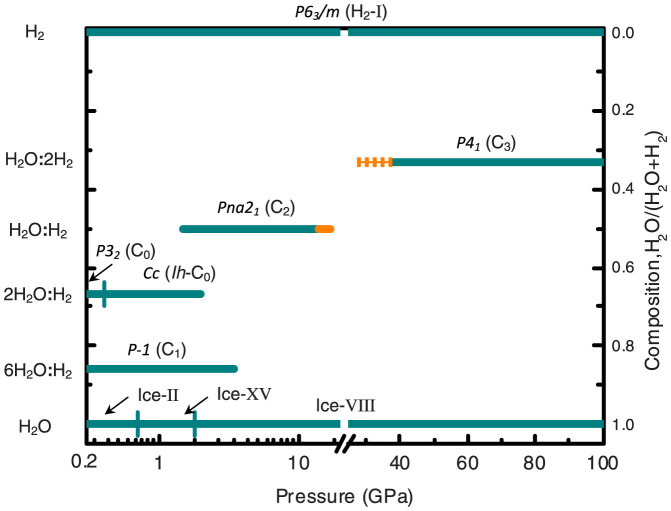
Phase diagram of the H_2_O–H_2_ system. The stability ranges of C_2_ and C_3_ phases are calculated with and without ZPE effect. The solid orange line represents extra stability range added due to ZPE, the dashed orange line represents regions that become unstable after inclusion of the ZPE.

**Figure 4:**
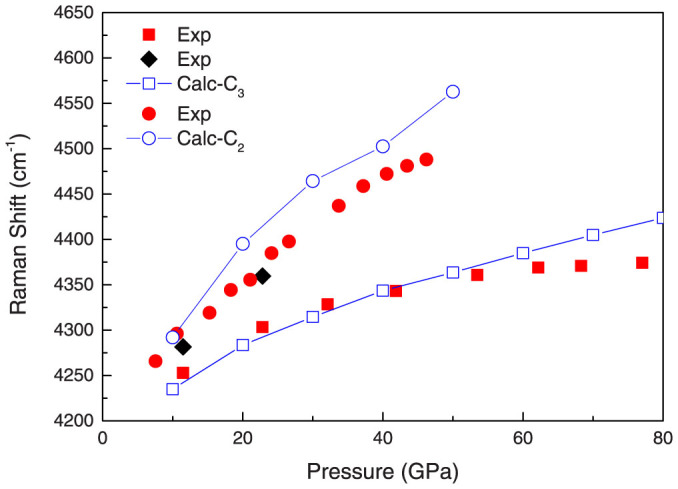
Variations of the Raman shift of the vibron for the H_2_ molecules with pressure from experimental data in Ref. [9] and our theoretical calculations. The red and black symbols are the experimental data for H_2_ vibrons in the H_2_-D_2_O sample. The blue open circles and squares indicate the Raman shift calculation for C_2_ and C_3_ phases of H_2_-D_2_O system, respectively.

The C_3_ phase starts to be energetically favorable above ~38 GPa when including ZPE, as shown in Fig. 3. Thus, the novel C_3_ phase can be synthesized in hydrogen-rich conditions at pressures starting from 38 GPa. This theoretical value agrees well with the experimentally observed formation at 45–50 GPa of a mysterious phase of unknown composition^7^,^10^. As shown in Fig. 4, calculations of the Raman shift^23^ reveal differences between the C_2_ and C_3_ phases in the H_2_-D_2_O system. The Raman shift of C_3_ phase, rather than an amorphous phase, agrees very well with the lower Raman frequencies of the vibron for the hydrogen molecules observed in Ref. [9]. The black rhombi in Fig. 4 indicate that some of the H_2_-D_2_O C_3_ sample encountered decomposition when quenched to low pressure. The variation of lattice parameters of the ice host structure in hydrates with pressure, revealed by our theoretical calculations, also agrees well with experimental XRD results^10^. At 55 GPa, our calculation gives lattice parameter of C_3_ phase *a* = *b* = 4.00 *Å* and *c* = 5.67 *Å*, corresponding to cubic ice sublattice with periodicity 5.67 *Å*, whereas experiment gives ~5.5 *Å*^10^.

At low pressure, the C_2_ adopts a “cubic ice” host structure and then transforms to a “tetragonal” one at ~20 GPa^10^ (Fig. S4 in Supplementary Information). When forming the C_3_ phase at increased pressure and in excess of H_2_, the ice host structure transforms to the “cubic ice” again. The change from tetragonal to “cubic” structure occurs before H-bond symmetrization transition happens in “tetragonal” type C_2_ at ~55 GPa. Thus, such structural transformation is unrelated to symmetrization of the H-bonds, but comes from the emergence of the C_3_ phase. For the hydrate C_3_, the H-bond symmetrization is predicted to occur at ~120 GPa (Fig. S5 in Supplementary Information), which is close the theoretical H-bond symmetrization pressure in ice-VII^14^.

## Discussion

The C_2_ and C_3_ hydrates have a similar ice host framework, but the different numbers of hydrogen molecules and their different locations and orientations bring huge differences in phase stability range. In the C_2_ phase, hydrogen molecules stay in the centers of cages formed by water molecules in contrast to C_3_ phase, where they are located at the faces of the cages. To clarify the causes of stability of hydrogen hydrates, we used Bader analysis^24^,^25^, and focused on the C_2_ and C_3_ phases (Fig. S6 in Supplementary Information). We found a very small charge transferred from H_2_ to water molecules, so that the H_2_ molecules are slightly positively charged, and H_2_O molecules carry a slight negative charge. The magnitudes of these charges are ~10^−3^–10^−2^ per molecule. This suggests that interactions between these molecules are almost purely steric, mainly related to packing density and shapes of the molecules. Comparing Bader volumes of the H_2_O and H_2_ molecules in the hydrates and in pure H_2_O and H_2_, we see that water molecules occupy slightly larger volume in the hydrates, whereas hydrogen molecules occupy much less space in C_3_ hydrate than in pure H_2_ – this leads to net densification, stabilizing this phase in a wide pressure range. For the C_2_ hydrate, the H_2_ molecules have lower volume than in pure H_2_ only at pressures below ~10 GPa, which explains its instability at higher pressures.

**Figure 5:**
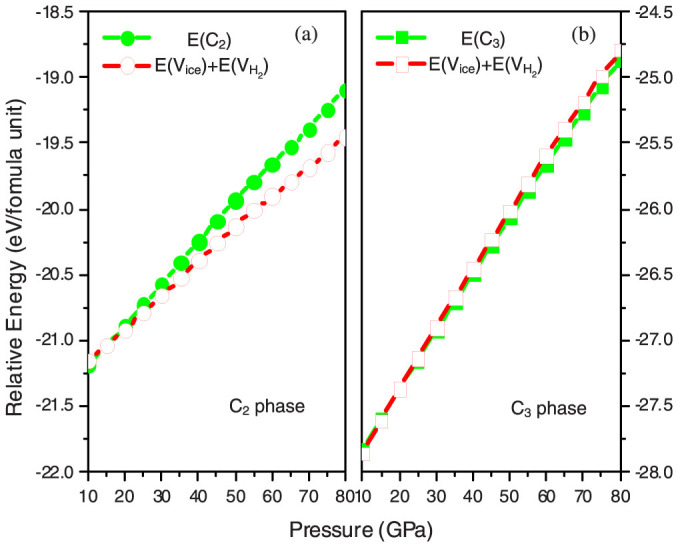
Internal energy of the C_2_ and C_3_ phases relative to the isochoric mixture of H_2_O and H_2_. Green lines represent the energy of the hydrate phases; red lines represent the energy of the isochoric mixture of ice-VIII and H_2_-I phases.

Having considered the *PV* -term in the enthalpy (*H* = *E* + *PV*), to get additional insight, we turned to the internal energy *E* and its changes when the H_2_ and H_2_O molecules are placed from the hydrate into pure H_2_ and H_2_O phases, while keeping molecular volumes fixed at their values in the hydrate (Fig. 5). This energy characterizes the net balance of the vdW attraction and steric repulsion between the molecules: this net effect is very small in the C_3_ phase (slightly destabilizing below ~30 GPa and slightly stabilizing above ~30 GPa). The remarkably wide stability field of the C_3_ phase is therefore mostly due to its high density and only to a small extent to more favorable intermolecular interactions. A much more interesting picture is observed for the C_2_ phase (Fig. 5a): we find its slight energetic stabilization below ~15 GPa, and an increasingly large destabilization at higher pressures. This explains why C_2_ is unstable at high pressures, and furthermore, it is clear that the increasing energetic instability of the C_2_ phase is responsible for the displacive phase transition, metastably occurring on overcompression and transforming the cubic H_2_O host sublattice into tetragonal, to enable better packing of the molecules.

Our calculations found that a C_3_-type phase is stable in the H_2_O–He system at 8–75 GPa (without including zero-point energy), and this phase is denser than the mixture of H_2_O and He. On the other hand, no such phase was found in the H_2_O-Ne system, and indeed the C_3_ phase is not packing-efficient in this system (Fig. S7 and Fig. S8 in Supplementary Information). He and Ne are both chemically extremely inert, their almost only differences are size and (here insignificant) mass. Stability of He-C_3_ and instability of Ne-C_3_ hydrates reinforce our conclusion made for the H_2_O–H_2_ system, that stability of this novel phase comes not from specific bonding interactions between the molecules, and not even their shapes, but is mostly due to their very efficient packing.

In summary, using the evolutionary algorithm USPEX, we explored the H_2_O–H_2_ system at pressures of up to 100 GPa. Stoichiometries and stability fields of H_2_O–H_2_ hydrate phases have been studied. A series of pressure-induced transformations found by theory closely coincides with experimental data, but also new insight was obtained. A novel *Ih*–C_0_ structure is predicted to have a very close enthalpy to the recently discovered C_0_ structure. At pressures above 38 GPa, novel hydrogen hydrate C_3_, based on cubic ice Ic, is predicted to be stable. With stoichiometry H_2_O:2H_2_, this is the hydrogen-richest hydrate known to date. With gravimetric density of easily removable hydrogen (18 wt.%), this is a promising hydrogen storage material that can find practical applications if its synthesis pressure can be decreased.

## Methods

### Crystal structure prediction

Predictions were done using the USPEX code in its variable-composition mode at several pressures (0, 1, 2, 5, 10, 20, 50 and 100 GPa) and zero temperature. A number of studies illustrate the power of the USPEX method^26^,^27^,^28^. We have done two types of variable-composition structure predictions in searching for all stable phases in the H–O system: (1) In the H–O system, assembling the structures from atoms, and (2) In the H_2_O–H_2_ system with giving H_2_O and H_2_ molecules as structure building blocks. We have found that, even in the H–O system, all low-enthalpy states at pressures of our interest (<120 GPa) are actually made of well-defined H_2_O and H_2_ molecules. This allowed us to focus on molecular-type calculations, capable of efficiently dealing with large systems, without loss of rigor.

Given molecular nature of all stable and nearly stable compounds in this system, we searched for the packing of well-defined H_2_O and H_2_ molecules (rather than H and O atoms), by applying the specially designed constrained global optimization algorithm, considering structures with up to 24 molecules (i.e. up to 72 atoms) per primitive unit cell.

### DFT calculations

Structure relaxations were done using density functional theory (DFT) within van der Waals (vdW) functional optB88-vdW^29^ in the framework of the all-electron projector augmented wave (PAW)^30^ method as implemented in the VASP^31^ code. The plane wave kinetic energy cutoff of 600 eV and Gamma-centered *k*-point meshes with the reciprocal space resolution of 2*π* × 0.05 *Å* were used. Having identified the most stable compositions and candidate structures, we relaxed them at pressures from 1 *atm* to 120 GPa with an even higher cutoff of 800 eV to refine their thermodynamic properties and stability fields. Structure relaxations proceeded until net forces on atoms were below 1 meV/*Å*, which gave us enthalpies converged to better than 1 meV/atom.

It is expected that the relative contribution of hydrogen bonding (H-bonding) and van der Waals (vdW) dispersion forces has a significant impact on the phase transition pressures and cohesive properties of the various crystalline ice phases^32^. This is also confirmed by our calculations for the phase transition pressures of ice phases from optB88-vdW, GGA^33^ calculations and experiments (Fig. S1 in Supplemental Information). Thus, all calculations included the vdW functional to treat the vdW forces, unless stated otherwise.

## Author Contributions

G.R.Q., A.O.L., Q.Z., A.R.O. and X.D. designed research, performed simulations, analyzed data, and wrote the manuscript.

## Supplementary Material

Supplementary Information Novel Hydrogen Hydrate Structures under Pressure
