# Supplementary material for: Novel Hydrogen Hydrate Structures under Pressure
Source: Sci Rep. 2014 Jul 8;4:5606. doi: 10.1038/srep05606 (PMC4085642; doi:10.1038/srep05606)
Supplement: Supplementary Information — Novel Hydrogen Hydrate Structures under Pressure [file srep05606-s1.pdf]

Novel Hydrogen Hydrate Structures under Pressure

Supplementary information

Guang-Rui Qian,[1] Andriy O. Lyakhov,[1] Qiang Zhu,[1] Artem R. Oganov,[2,3,4] and Xiao Dong[1,5]

[1]*Department of Geosciences, Stony Brook University, Stony Brook, New York 11794-2100, USA*
[2]*Department of Geosciences, Center for Materials by Design, and Institute for Advanced Computational Science, State University of New York, Stony Brook, NY 11794-2100*
[3]*Moscow Institute of Physics and Technology, 9 Institutskiy lane, Dolgoprudny city, Moscow Region 141700, Russia*
[4]*School of Materials Science, Northwestern Polytechnical University, Xi'an 710072, China*
[5]*School of Physics and MOE Key Laboratory of Weak-Light Nonlinear Photonics, Nankai University, Tianjin 300071, China*

Phase	Space group # atoms	Pressure (GPa)	Lattice parameters (Å, °)			Atomic coordinates (fractional)			
Ih-C_0	Cc	0.5	a=4.540	b=7.913	c=6.893	H1	0.3164	0.0216	0.2756
	24		α=90.00	β=90.43	γ=90.00	H2	-0.0836	0.4759	0.2074
						H3	0.0368	0.2234	0.0087
						H4	-0.1425	0.0458	0.0087
						H5	-0.1533	0.1675	0.2993
						H6	0.0312	0.2282	0.4859
						O1	-0.1503	0.1671	0.0507
						O2	-0.1512	0.1655	0.4451
C_0	$P3_2$	0.5	a=6.054	b=6.054	c=6.044	H1	-0.2342	0.2468	0.2423
	24		α=90.00	β=90.00	γ=120.0	H2	-0.2052	0.1995	0.3471
						H3	-0.2848	-0.2943	0.4177
						H4	-0.1054	-0.0865	-0.3963
						H5	0.3232	-0.2477	-0.1620
						H6	-0.3904	-0.0361	-0.0915
						O1	-0.1065	-0.1969	0.4788
						O2	0.4309	-0.1422	-0.0335
$Pna2_1$-C_2	$Pna2_1$	5.0	a=4.325	b=6.213	c=4.353	H1	-0.0633	-0.1716	0.0980
	20		α=90.00	β=90.00	γ=90.0	H2	0.0324	-0.0823	0.0346
						H3	-0.1927	0.28048	0.06532
						H4	0.0034	-0.4665	-0.2512
						O1	-0.0038	0.3755	0.0573
$I4_1md$-C_2	$I4_1md$	5.0	a=4.397	b=4.397	c=6.055	H1	0.0000	-0.4153	-0.0492
	10		α=90.00	β=90.00	γ=90.0	H2	0.0000	-0.1840	0.2928
						O1	0.0000	0.0000	0.1935
$P4_12_12$-C_2	$P4_12_12$	20.0	a=3.987	b=3.987	c=5.801	H1	-0.2224	0.3043	-0.2996
	20		α=90.00	β=90.00	γ=90.0	H2	0.2445	0.03610	-0.3953
						O1	-0.2458	-0.2458	0.00000
$I4_1/amd$-C_2	$I4_1/amd$	40.0	a=3.604	b=3.604	c=5.932	H1	0.0000	0.0000	0.0000
	10		α=90.00	β=90.00	γ=90.0	H2	0.0000	0.2500	0.4366
						O1	0.0000	0.7500	0.1250
C_3	$P4_1$	40.0	a=4.117	b=4.117	c=5.850	H1	0.3125	0.0251	0.1689
	28		α=90.00	β=90.00	γ=90.00	H2	-0.2083	-0.4287	-0.3718
						H3	0.1920	-0.0158	0.0823
						H4	-0.2936	0.4173	-0.3779
						H5	0.2548	-0.0448	-0.4054
						H6	-0.2549	-0.4575	0.1542
						O	-0.2518	-0.2499	0.2497

TABLE S1. Structures of the predicted hydrogen hydrates. The numbers of atoms in the primitive cell are given.

[1] Salzmann, C. G., Radaelli, P. G., Mayer, E. & Finney, J. L. Ice xv: A new thermodynamically stable phase of ice. *Phys. Rev. Lett.* **103**, 105701 (2009).
[2] Fletcher, N. H. *The Chemical Physics of Ice* (Cambridge University Press, 1970).
[3] Pickard, C. J. & Needs, R. J. Structure of phase III of solid hydrogen. *Nature Physics* **3**, 473–476 (2007).

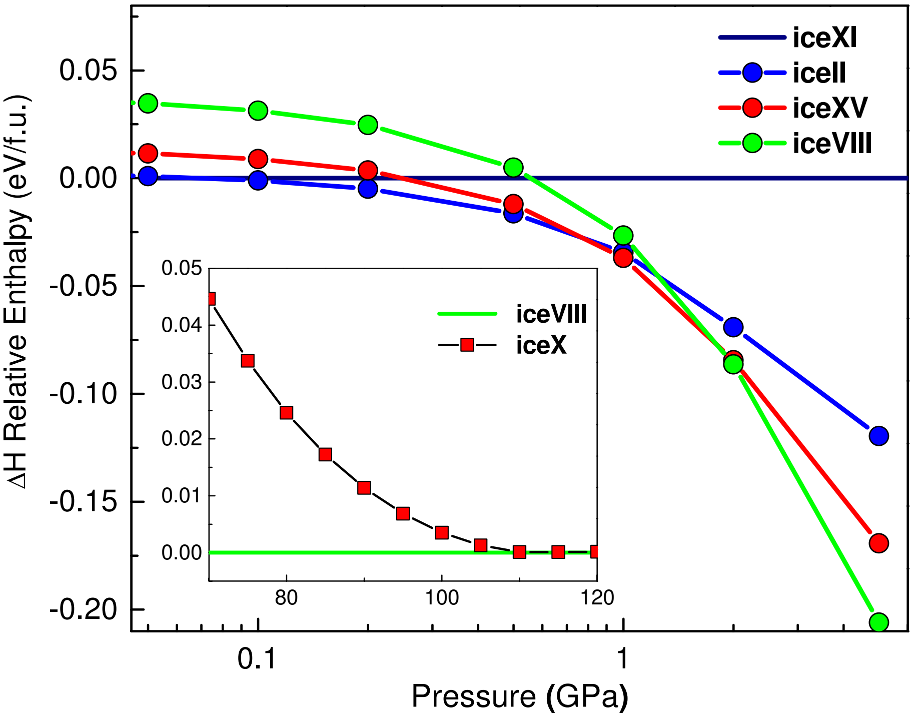

FIG. S1. (Coloronline) Enthalpy relative to ice XI as a function of pressure with a van der Waals functional. The phase transition sequence at T = 0 K is ice XI → II → XV → VIII → X. Tthe transition pressures are 2.4/0.1/0.2 GPa, 3.6/0.7/0.6 GPa, 6.5/1.8/2.1 GPa and 100/110/90 GPa from DFT with no vdw/DFT+vdw/experiments.[1,2]

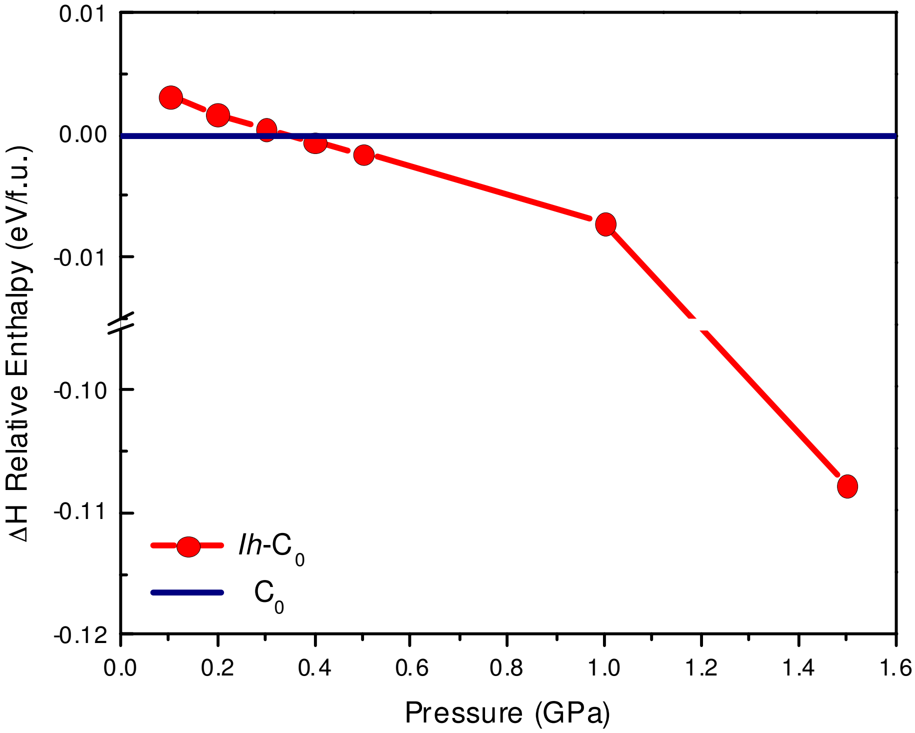

FIG. S2. (Color online) Enthalpies of the Ih-C_0 phase relative to C_0 phase as a function of pressure. The Ih-C_0 phase is more energetically favorable above ~0.4 GPa than the C_0 phase.

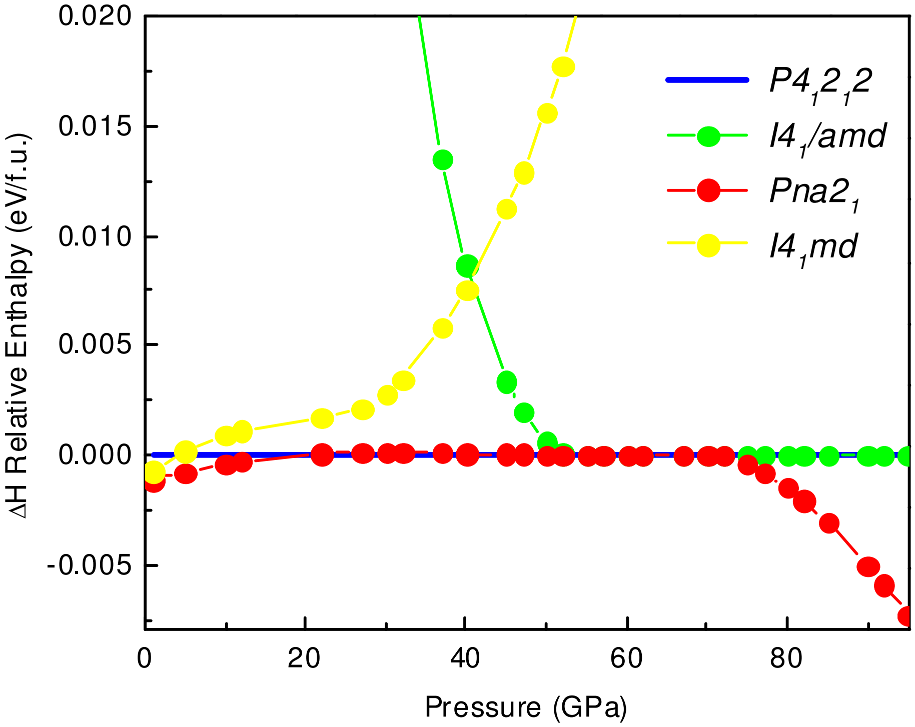

FIG. S3. (Color online) Enthalpy of $I4_1/amd$, $Pna2_1$ and $I4_1md$ variants of the C_2 structure relative to the $P4_12_12$ structure as a function of pressure. Near 70 GPa, the $Pna2_1$ H_2O-H_2 transforms to the *Imma* phase.

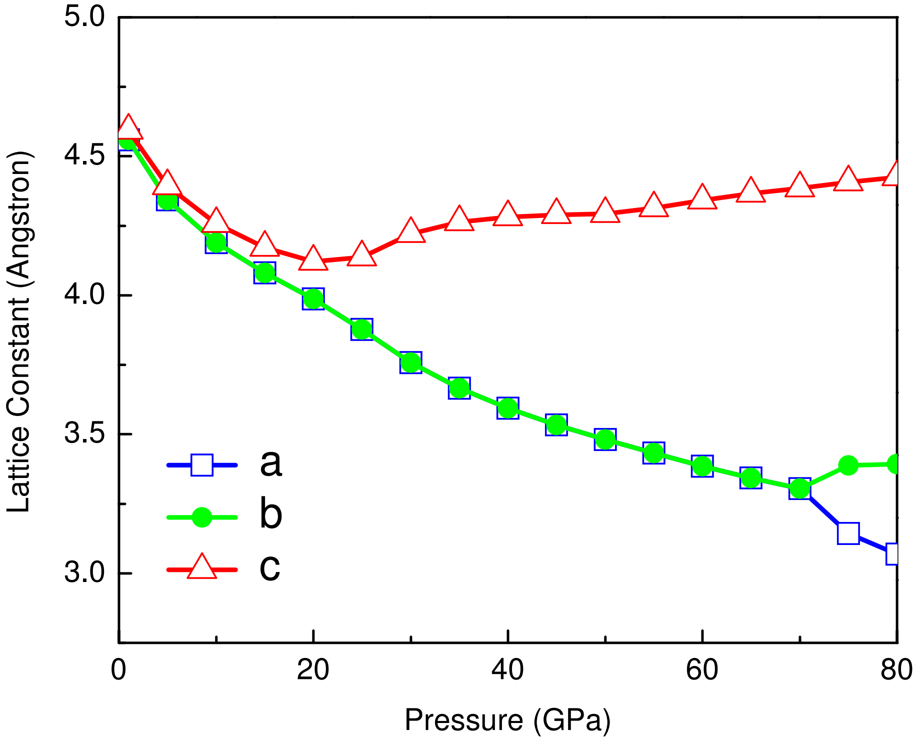

FIG. S4. (Color online) Lattice parameters in $Pna2_1$ type C_2 as a function of pressure. The "cubic" ice framework transforms into tetragonal structure near 20 GPa, then transforms into an orthorhombic structure around 70 GPa.

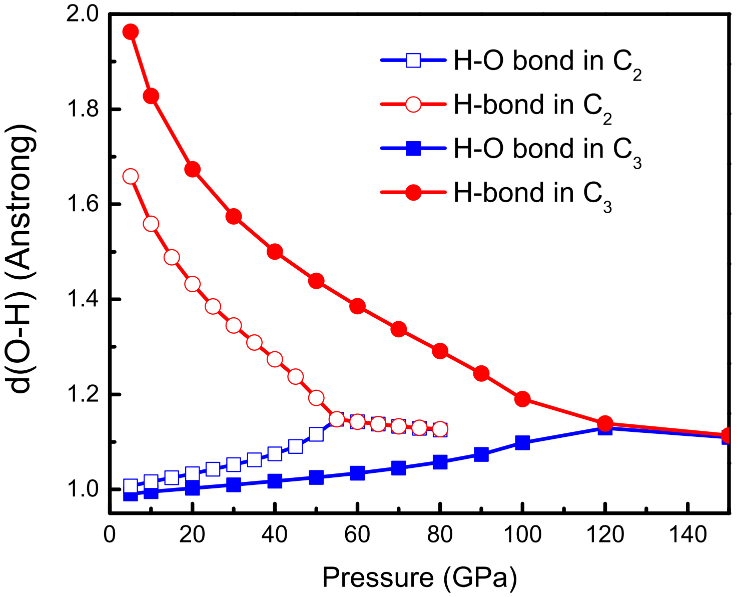

FIG. S5. (Color online) The H-O distances in C_2 and C_3 phases. The H-bond symmetrization happens around 55 GPa in the C_2 phase and around 120 GPa in the C_3 phase.

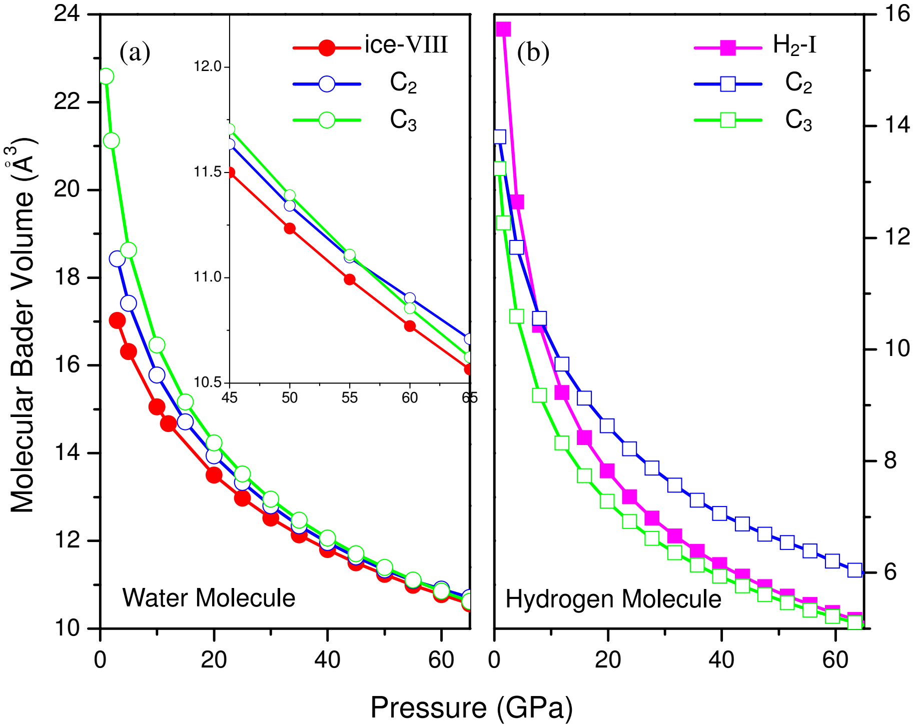

FIG. S6. (Color online) Bader volumes of the water and hydrogen molecules in ice-VIII, H_2-I[3], C_2 and C_3 phases as a function of pressure.

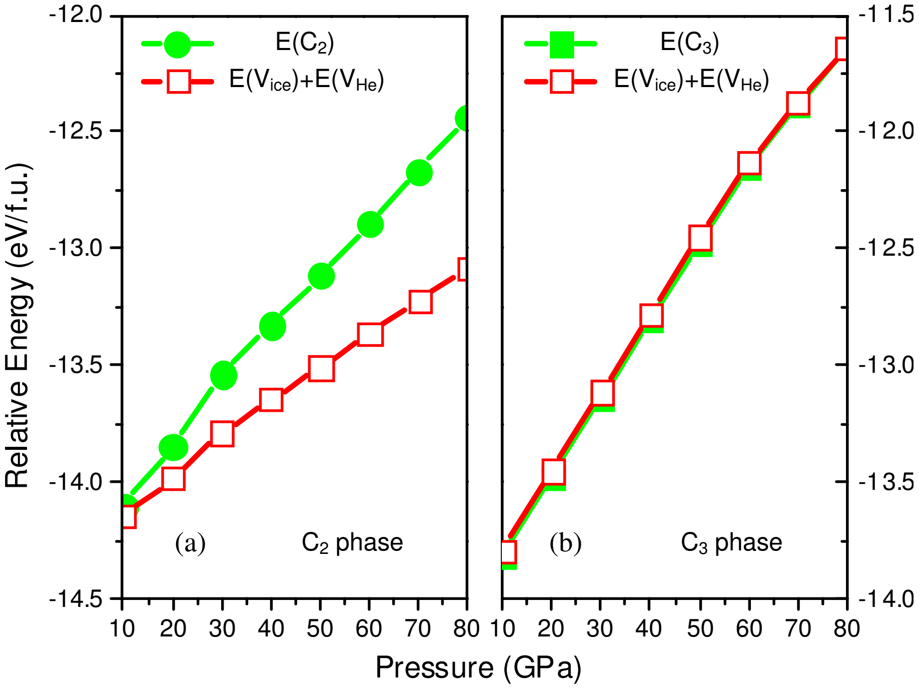

FIG. S7. (Color online) Internal energy of the C_2 and C_3 phases at neon hydrates relative to the isochoric isochemical mixture of H_2O and He. Green lines represent the energy of the hydrate phases; red lines – the energy of the isochoric isochemical mixture of ice-VIII and He hcp phases.

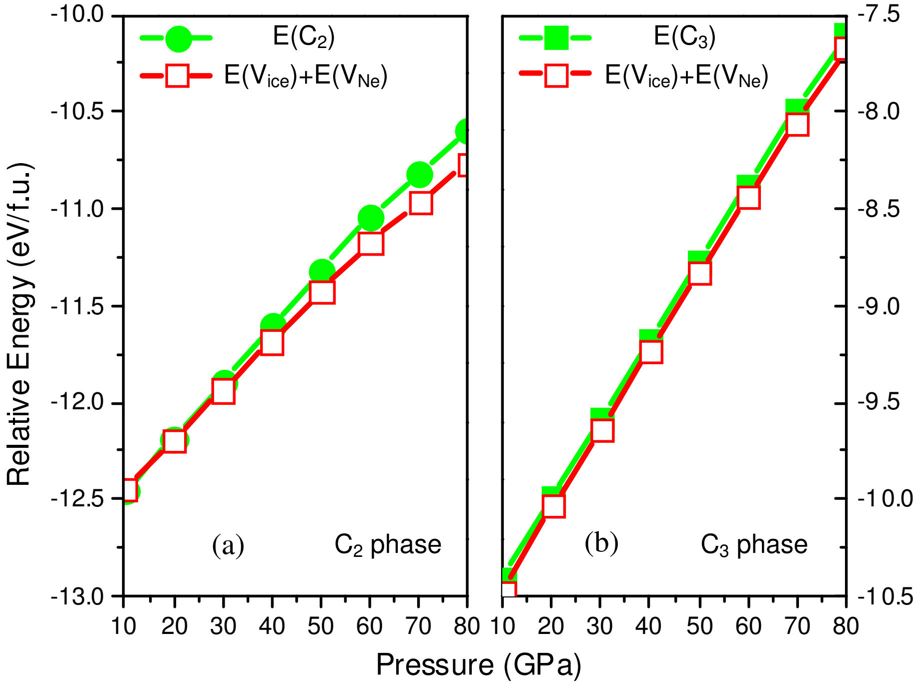

FIG. S8. (Color online) Internal energy of the C_2 and C_3 phases at neon hydrates relative to the isochoric isochemical mixture of H_2O and Ne. Green lines represent the energy of the hydrate phases; red lines – the energy of the isochoric isochemical mixture of ice-VIII and Ne hcp phases.

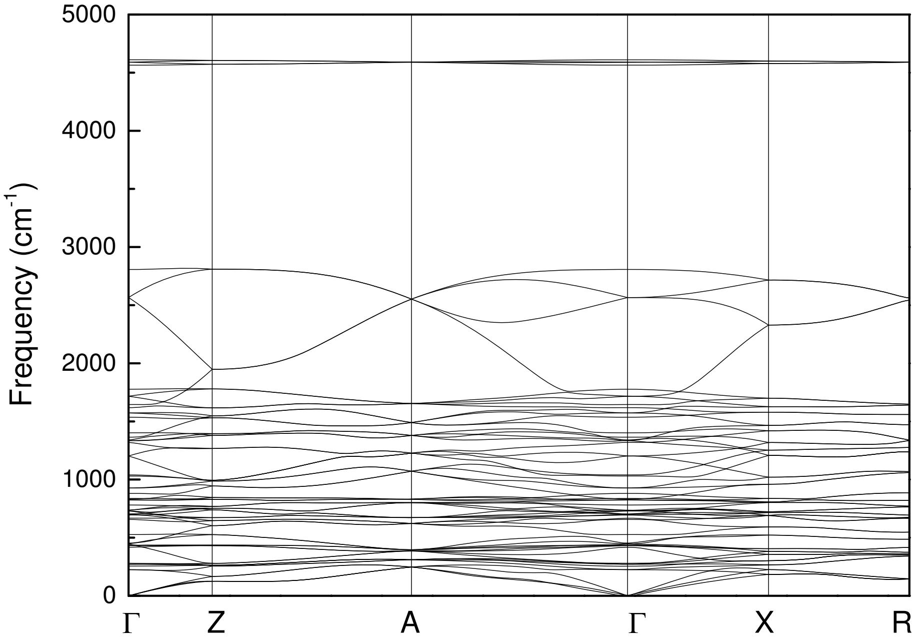

FIG. S9. (Color online) Phonon dispersion curves of the $P4_12_12$ type C_2 phase at 40 GPa.

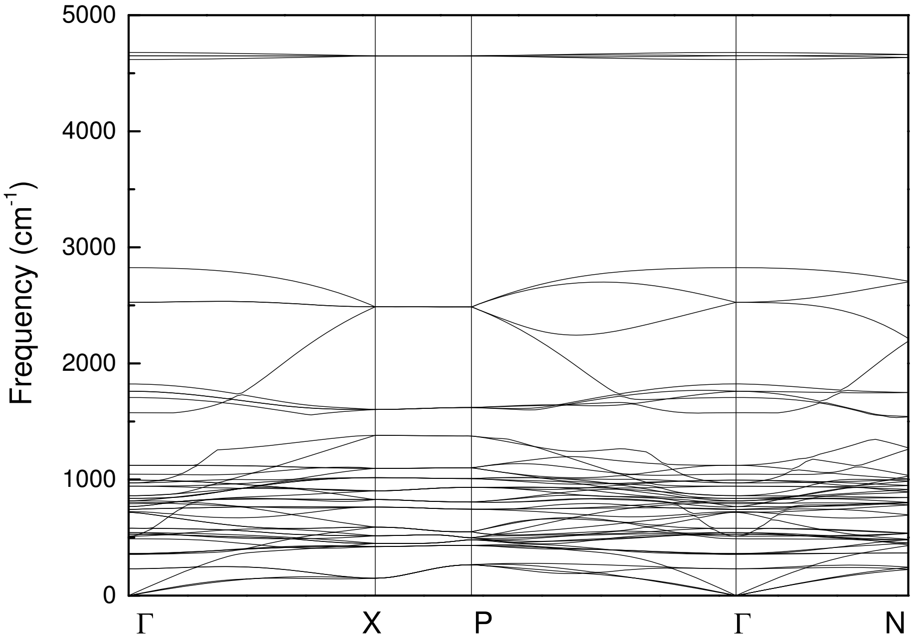

FIG. S10. (Color online) Phonon dispersion curves of the $I4_1/amd$ type C_2 phase at 60 GPa.

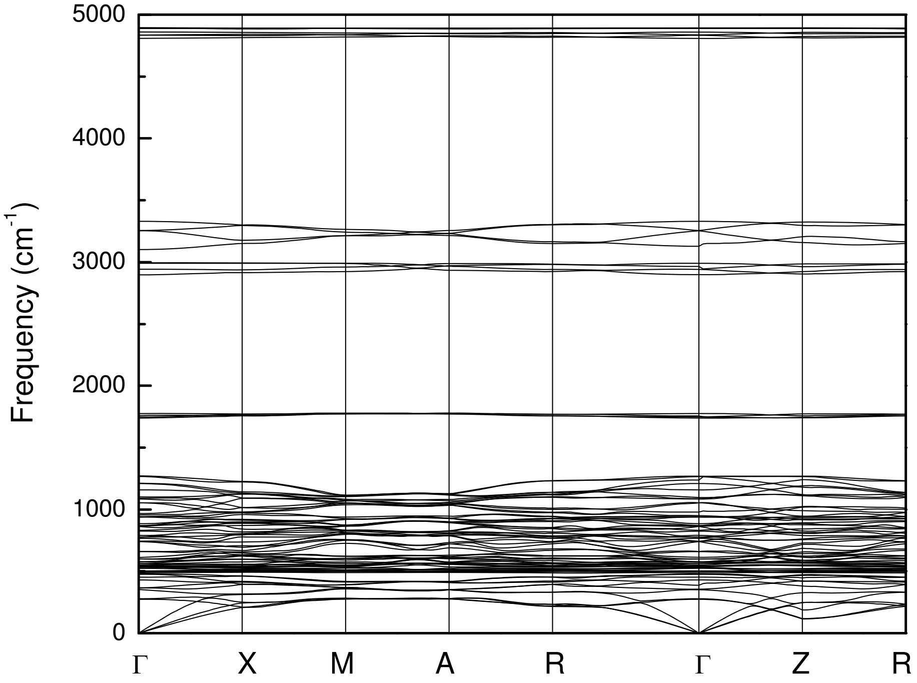

FIG. S11. (Color online) Phonon dispersion curves of the C_3 phase at 40 GPa.

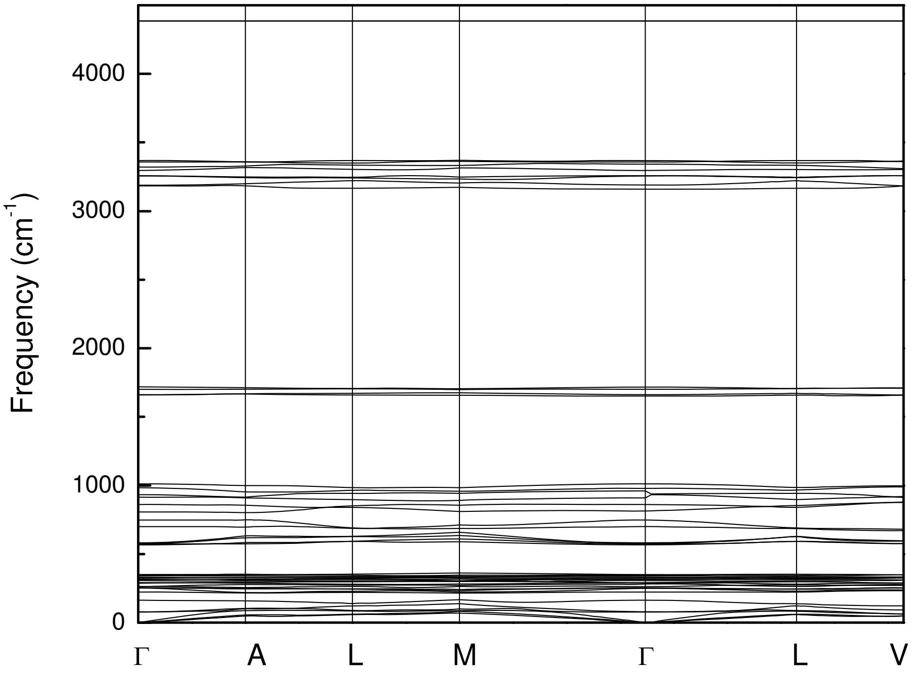

FIG. S12. (Color online) Phonon dispersion curves of the Ih-C_0 phase at 0.4 GPa.
